# Incidence and outcomes of diabetes mellitus amongst hospital admissions with *de novo* heart failure; a retrospective cohort study from a nationwide registry

**DOI:** 10.3389/fcdhc.2026.1719223

**Published:** 2026-03-06

**Authors:** Ammar Chapra, Rasha Kaddoura, Ashfaq Patel, Tina Francis, Sharin Varghese, Mohamed Izham Mohamed Ibrahim, Jassim Shah, Haisam Alsadi, Abdelfatah Elasfar, Amr Badr, Rajvir Singh, Abdulrahman Arabi

**Affiliations:** 1 Department of Cardiology, Heart Hospital, Hamad Medical Corporation, Doha, Qatar; 2 College of Pharmacy, Qatar University Health Sector, Qatar University, Doha, Qatar; 3 Medical Research Center, Hamad General Hospital, Doha, Qatar

**Keywords:** prediabetes, acute heart failure, *de novo* heart failure, diabetes mellitus, metabolic screening

## Abstract

**Background:**

Diabetes Mellitus (DM) is a recognized risk factor for the development of acute and chronic heart failure (HF). *De Novo* HF (DNHF), defined as acute HF occurring in patients with no prior history of HF, is a distinct type which is usually seen in patients with established cardiovascular risk factors such as DM. However, in some cases, DNHF may be the initial manifestation of DM and lead to a new diagnosis of DM during admission for HF symptoms. The incidence of this occurrence is under-recognized in the setting of DNHF. Our study aimed to determine the incidence of newly diagnosed DM in patients presenting with DNHF and to determine any differences in clinical characteristics or short-term outcomes across the spectrum of DM.

**Methods:**

This retrospective cohort study was conducted at the largest advanced HF center in Qatar. All patients admitted with DNHF were grouped as: 1) newly diagnosed DM, 2) pre-existing DM, 3) prediabetes, and 4) no DM. Clinical characteristics and in-hospital/post-discharge outcomes were described. Continuous variables were reported as mean with standard deviation or IQR and categorical variables as frequency distribution with percentage.

**Results:**

Among 260 patients with DNHF, 173 (66.5%) had DM, and 52 (20%) were prediabetic. Newly diagnosed DM was identified in 26 (10%), 147 (56.5%) were known diabetics. Compared with known DM, newly diagnosed patients were predominantly male (92.3% *vs*. 74.1%), had fewer comorbidities such as hypertension (84.6% *vs*. 90.5%), coronary artery disease (57.7% *vs* 79.6%), chronic kidney disease (11.5% *vs*. 29.9%), had a greater non-ischemic etiology of HF (30.4% *vs*. 23.2%), and higher prevalence of HFrEF (92.3% *vs* 70.3%). In-hospital mortality and readmissions were similar across all groups.

**Conclusion:**

Our findings indicate the high burden of dysglycemia (DM and prediabetes) that is present in DNHF patients. Furthermore, DNHF served as the initial clinical presentation of DM or prediabetes in approximately one-third of our patients. This underscores the need for routine metabolic screening in patients presenting with DNHF, as early identification and management of DM is crucial in this population to improve outcomes.

## Graphical Abstract

**Figure:**
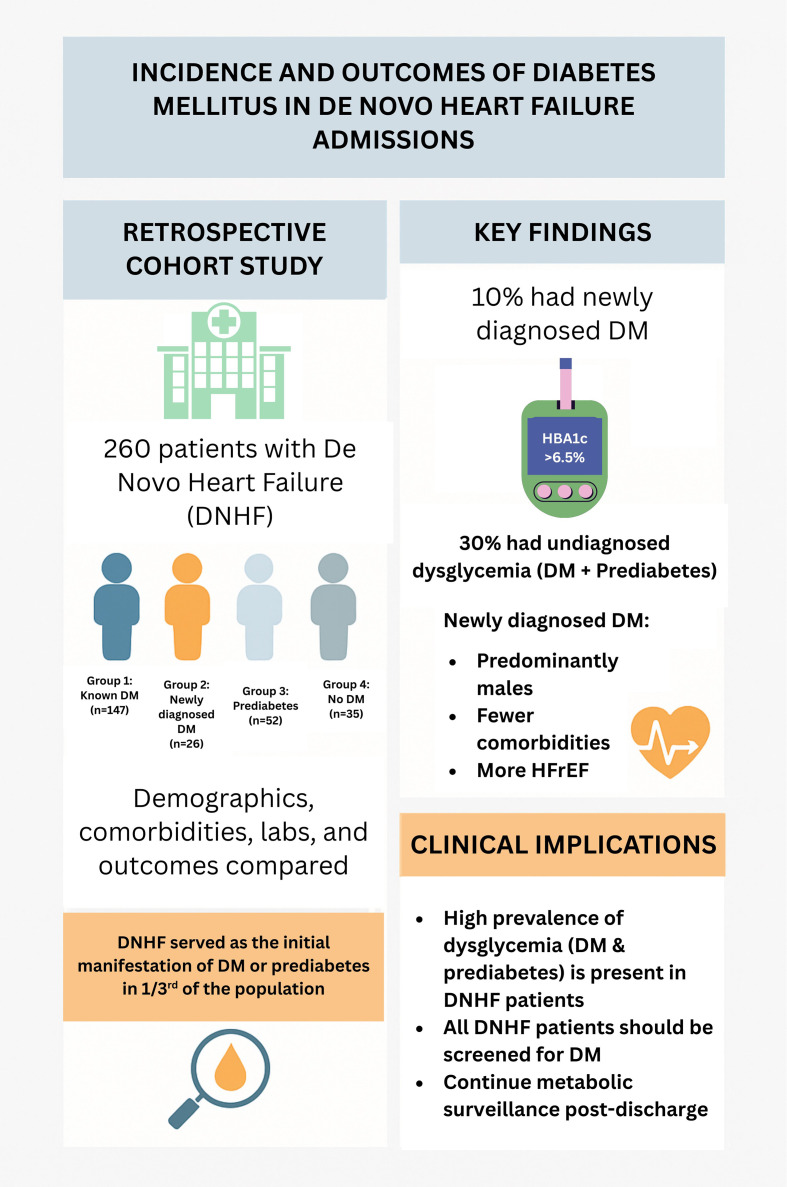

## Highlights

What is currently known about this topic?

- Diabetes is a strong risk factor for heart failure (HF)
- *De Novo* HF (DNHF) may be the initial presentation of Diabetes Mellitus (DM)

What is the key research question?

- What is the incidence and clinical profile of newly diagnosed diabetes in patients with DNHF?

What is new?

- This study identified that one in ten patients with DNHF were newly discovered to have DM during hospital admission for HF.
- One in three patients had previously unrecognized dysglycemia (DM and prediabetes).
- Nearly half of those with prediabetes developed overt DM on a median of 26 months of follow-up.

How might this study influence clinical practice?

- These findings support routine screening for DM with HBA1c upon admission with DNHF and longitudinally on follow-up visits.

## Background

Acute heart failure (AHF) is a clinical syndrome characterized by the sudden onset of worsening signs and symptoms of heart failure (HF), typically driven by elevated cardiac filling pressures and systemic congestion. AHF presentations are broadly categorized into two phenotypes: *de novo* HF (DNHF) and acutely decompensated chronic HF (ADCHF). DNHF refers to the first clinical presentation of HF in individuals without a prior diagnosis of HF, whereas ADCHF involves a sudden worsening of symptoms in patients with established chronic HF. DNHF is often precipitated by acute cardiac events such as myocardial infarction or severe hypertension and is less frequently associated with chronic comorbidities (1, 2). However, in some cases, DNHF may be the only initial presentation for undiagnosed cardiovascular risk factors such as Diabetes Mellitus (DM) (3, 4). DM, especially type 2 DM, is a major risk factor for both acute and chronic HF. The pathophysiological mechanisms by which DM may lead to HF include diabetic cardiomyopathy, characterized by myocardial fibrosis and impaired relaxation, and by contributing to other risk factors for HF such as coronary artery disease and hypertension (5). Conversely, HF can exacerbate insulin resistance and glucose metabolism abnormalities, creating a bidirectional relationship between these conditions (6, 7). This interplay is well-established in chronic HF, where the prevalence of DM is consistently high (8). However, the burden of newly diagnosed DM in patients presenting with DNHF is less well studied. Hospitalization for DNHF may serve as the initial manifestation of abnormal glucose metabolism, possibly due to the physiological stress response or because inpatient evaluations uncover previously undiagnosed prediabetes or DM. It is critical to understand the burden of this silent *dysglycemia* (DM or prediabetes), due to its implications for primary prevention strategies and early intervention. This is particularly relevant in Qatar, where the prevalence of type 2 DM is projected to double by the year 2050 (9). This study aimed to determine the incidence of newly diagnosed DM in patients admitted with DNHF and to compare their clinical characteristics and short-term outcomes with those of patients with known DM, prediabetes, or normoglycemia.

## Methods

This retrospective observational study was conducted at the Heart Hospital, the largest cardiology center in Qatar. The study included adult patients with DNHF who are enrolled in the Heart Failure Clinical Registry at the Heart Hospital. The registry recruited patients admitted with a principal diagnosis of acute heart failure. The registry was approved by the Institutional Review Board of the Heart Hospital and the Medical Research Center (MRC-01-22-631). A waiver of informed consent was granted due to the retrospective nature of recruitment. The study was conducted according to the principles of the Declaration of Helsinki, Good Clinical Practice, and within the laws and regulations of the Ministry of Public Health in Qatar. This study only included adult patients diagnosed with DNHF who were admitted between December 2017 and January 2024. Patients who developed heart failure during their hospital stay were excluded. Patients were categorized into four groups: patients with pre-existing DM, patients with newly diagnosed DM during hospital admission, patients with newly diagnosed prediabetes during hospital admission, and patients without DM. All patient-related data was collected through electronic medical records (Cerner^®^). The collected data included demographics, baseline characteristics, treatment, and clinical outcomes. A diagnosis of DM was based on a single HbA1c reading which was collected within 24 hours of admission as part of the initial lab investigations which is the standard of care at our institution. To understand the progression of DM, HbA1c data was collected among those with available repeat HbA1c upon follow-up. The reported outcomes included in-hospital death during index admission, readmissions, and length of hospital stay. The follow-up period was defined as the time from hospital discharge to the end of the study period or last known contact in our electronic health records.

### Statistical analysis

The de-identified data were exported from the electronic medical records to Excel sheet then transferred to statistical software (IBM Corp. Released 2021. IBM SPSS Statistics for Windows, Version 29.0. Armonk, NY: IBM Corp). The sample size calculation was not attempted as all the patients who were admitted in the specified study duration were included. The numerical or continuous variables were expressed as mean and standard deviation or Inter Quartile Range (IQR) as appropriate. Categorical variables were expressed as frequency distribution with percentage. One Way Analysis of Variance (ANOVA) and Kruskal Walli’s tests were used to compare the continuous variables wherever applicable within DNHF categories and chi-squared tests were used for categorical variables. A two-tailed p-value of less than 0.05 was considered statistically significant level.

## Results

### Patient characteristics

Within the specified study duration, 710 patients included in the registry were screened. Of them 260 (36.6%) patients were diagnosed with DNHF and the remaining 450 (63.4%) patients with chronic HF. Among the patients with DNHF, there were 147 (56.5%) patients with a history of DM prior to admission, whereas 26 (10.0%) patients were newly diagnosed with DM during hospital admission (Central Illustration). Prediabetics accounted for 20.0% (n=52) and non-diabetics for 13.5% (n=35) of patients. Patients with new and known diabetes were older (60.3 and66.6 years) than prediabetics (57.6 years) and non-diabetics (52.3 years). The proportion of active smokers were higher among the prediabetic and non-diabetic groups (25.0% and 39.4%) than among the new and known diabetic groups (9.5% and 15.0%). Males accounted for most of the study population (range across the groups: 74.1 – 92.3%). Diabetics (new and known) had significantly higher rates of hypertension (84.6% and 90.5%), dyslipidemia (69.2% and78.9%), and coronary artery disease (57.7% and 79.6%) than pre- and non-diabetics [(65.7 and 67.3%), (51.4 and 53.8%), and (34.6% and 40.0%), respectively]. Patients with known DM had significantly higher rates of undergoing percutaneous coronary intervention (34.0%), chronic kidney disease (29.9%), and chronic lung disease (22.4%) than the other groups. Unsurprisingly, prescriptions at baseline for renin-angiotensin-aldosterone system inhibitors (51.0%), diuretics (44.2%), antiplatelet therapy (32.0%), and diabetic medications (61.2%) were significantly higher in the known DM group than in the other groups. **Table 1** demonstrates the details of baseline characteristics.

**Table 1:** Baseline characteristics of patients with *de novo* heart failure.

Variable	Known DM (n=147)	New DM (n=26)	Pre-DM (n=52)	No DM (n=35)	P-value
Demographics					
Age (years)	66.6 ± 13.3 (n=147)	60.3 ± 13.8 (n=26)	57.6 ± 15.5 (n=52)	52.3 ± 17.9 (n=35)	0.001
Gender					
Females	38/147 (25.9%)	2/26 (7.7%)	8/52 (15.4%)	9/35 (25.7%)	0.110
Males	109/147 (74.1%)	24/26 (92.3%)	44/52 (84.6%)	26/35 (74.3%)	
Body mass index (kg/m2)	30.5 ± 14.3 (n=143)	28.2 ± 6.9 (n=26)	26.7 ± 5.6 (n=143)	34.9 ± 52.2 (n=34)	0.380
Smoking status					
Current smoker	20/133 (15.0%)	2/21 (9.5%)	12/48 (25.0%)	13/33 (39.4%)	0.003
Ex-smoker	24/133 (18.0%)	9/21 (42.9%)	7/48 (14.6%)	4/33 (12.1%)	
Non-smoker	89/133 (66.9%)	10/21 (47.6%)	29/48 (60.4%)	16/33 (48.5%)	
Comorbidities					
Hypertension	133/147 (90.5%)	22/26 (84.6%)	35/52 (67.3%)	23/35 (65.7%)	0.001
Dyslipidemia	116/147 (78.9%)	18/26 (69.2%)	28/52 (53.8%)	18/35 (51.4%)	0.001
Coronary artery disease	117/147 (79.6%)	15/26 (57.7%)	18/52 (34.6%)	14/35 (40.0%)	0.001
Previous myocardial infarction	20/147 (13.6%)	5/26 (19.2%)	5/52 (9.6%)	6/35 (17.1%)	0.630
Previous PCI	50/147 (34.0%)	3/26 (11.5%)	7/52 (13.5%)	4/35 (11.4%)	0.001
Previous CABG	12/147 (8.2%)	1/26 (3.8%)	1/52 (1.9%)	0/35 (0.0%)	0.140
Valve surgery	4/147 (2.7%)	0/26 (0.0%)	2/52 (3.8%)	0/35 (0.0%)	0.550
Atrial fibrillation	15/147 (10.2%)	0/26 (0.0%)	7/52 (13.5%)	3/35 (8.6%)	0.290
History of cardiac arrest	3/147 (2.0%)	1/26 (3.8%)	0/52 (0.0%)	0/35 (0.0%)	0.470
CVA/TIA	7/147 (4.8%)	0/26 (0.0%)	1/52 (1.9%)	1/35 (2.9%)	0.560
Anemia	23/147 (15.6%)	0/26 (0.0%)	8/52 (15.4%)	5/35 (14.3%)	0.200
Chronic kidney disease	44/147 (29.9%)	3/26 (11.5%)	5/52 (9.6%)	5/35 (14.3%)	0.005
Asthma/COPD	33/147 (22.4%)	1/26 (3.8%)	7/52 (13.5%)	1/35 (2.9%)	0.007
Medications and devices at baseline					
ACEI/ARB	75/147 (51.0%)	8/26 (30.8%)	17/52 (32.7%)	9/35 (25.7%)	0.010
Beta-blockers	39/147 (26.5%)	2/26 (7.7%)	13/52 (25.0%)	4/35 (11.4%)	0.060
Mineralocorticoids	6/147 (4.1%)	2/26 (7.7%)	2/52 (3.8%)	0/35 (0.0%)	0.480
HDZ/ISDN	3/147 (2.0%)	1/26 (3.8%)	0/52 (0.0%)	0/35 (0.0%)	0.470
Diuretics	65/147 (44.2%)	8/26 (30.8%)	16/52 (30.8%)	6/35 (17.1%)	0.020
Digoxin	3/147 (2.0%)	0/26 (0.0%)	0/52 (0.0%)	0/35 (0.0%)	0.510
Ivabradine	3/147 (2.0%)	2/26 (7.7%)	0/52 (0.0%)	0/35 (0.0%)	0.190
Antiplatelet therapy	47/147 (32.0%)	2/26 (7.7%)	7/52 (13.5%)	3/35 (8.6%)	0.001
Oral anticoagulation	40/147 (27.2%)	6/26 (23.1%)	13/52 (25.0%)	3/35 (8.6%)	0.140
Warfarin	6/147 (4.1%)	1/26 (3.8%)	5/52 (9.6%)	0/35 (0.0%)	0.190
NOACs	43/147 (29.3%)	5/26 (19.2%)	13/52 (25.0%)	5/35 (14.3%)	0.270
Diabetic medications	90/147 (61.2%)	5/26 (19.2%)	4/52 (7.7%)	1/35 (2.9%)	0.001
ICD/CRT/Pacemaker	5/147 (3.4%)	0/26 (0.0%)	3/52 (5.8%)	0/35 (0.0%)	0.350

ACEI/ARB, Angiotensin-converting enzyme inhibitors/Angiotensin II receptor blockers; CABG, coronary artery bypass graft; COPD, chronic obstructive pulmonary disease; CRT: cardiac resynchronization therapy; CVA, cerebrovascular accident; DM: diabetes mellitus; HDZ/ISDN, hydralazine/isosorbide dinitrate; ICD, implantable cardioverter-defibrillator; NOACs, non-vitamin K oral anticoagulants; PCI, percutaneous coronary intervention; TIA, transient ischemic attack.

### Hospital admission and discharge

The commonest physical exam findings of HF upon hospital admission were rales (range across the groups: 47.3 – 70.5%), with a raised jugular venous pressure manifested in 10.5 – 26.7% of all patients without a statistical difference between the study groups. Almost 40.0% of the patients presented with lower limb edema (range across the groups: 40 – 37.1%) and less than 10.0% of the patients had ascites (range across the groups: 2.3 – 7.1%). Vital signs and laboratory blood tests were comparable between the study groups. The mean natriuretic peptide levels among the groups ranged between 4443 and 7618 pg/mL and the mean glycated hemoglobin (HbA1c) was 8.3% and 8.7% in the known diabetics and newly diagnosed diabetics, respectively (**Table 2**).

**Table 2:** Physical examination, vital signs and laboratory tests upon hospital admission.

Variable	Known DM (n=147)	New DM (n=26)	Pre-DM (n=52)	No DM (n=35)	P-value
Signs of heart failure					
Rales	61/129 (47.3%)	12/23 (52.2%)	31/44 (70.5%)	16/32 (50.0%)	0.070
Raised JVP	11/79 (13.9%)	4/15 (26.7%)	6/29 (20.7%)	2/19 (10.5%)	0.500
Lower limb edema	57/142 (40.1%)	9/25 (36.0%)	20/50 (40.0%)	13/35 (37.1%)	0.970
Ascites	3/117 (2.6%)	1/26 (4.5%)	1/44 (2.3%)	2/35 (7.1%)	0.630
Vital signs					
Heart rate (bpm)	88.4 ± 20.2 (n=142)	97.7 ± 21.5 (n=26)	90.6 ± 23.0 (n=51)	89.4 ± 21.9 (n=34)	0.230
Systolic BP – supine (mmHg)	126.7 ± 24.8 (n=146)	135.0 ± 26.2 (n=26)	141.3 ± 140.9 (n=52)	128.2 ± 23.3 (n=35)	0.580
Diastolic BP – supine (mmHg)	75.7 ± 15.2 (n=146)	86.1 ± 16.8 (n=26)	77.8 ± 16.3 (n=52)	80.2 ± 23.0 (n=35)	0.030
Respiratory rate (bpm)	21.6 ± 9.9 (n=139)	20.4 ± 5.3 (n=24)	24.0 ± 15.7 (n=48)	19.5 ± 3.4 (n=34)	0.240
SpO2 – room air (%)	97.0 ± 2.1 (n=59)	98.4 ± 1.5 (n=14)	94.0 ± 15.8 (n=25)	97.9 ± 1.6 (n=21)	0.200
SpO2 – on oxygen (%)	89.1 ± 25.0 (n=64)	85.9 ± 29.7 (n=10)	84.9 ± 33.5 (n=22)	96.6 ± 2.7 (n=11)	0.660
Laboratory blood tests					
Hemoglobin (g/dL)	12.5 ± 2.3 (n=147)	14.1 ± 2.0 (n=26)	13.2 ± 2.3 (n=52)	12.9 ± 2.2 (n=35)	0.010
White blood cells (x 103 uL)	16.7 ± 73.2 (n=146)	11.3 ± 4.5 (n=26)	9.9 ± 4.4 (n=52)	9.3 ± 3.6 (n=35)	0.820
Neutrophil (%)	69.5 ± 30.1 (n=144)	65.5 ± 19.2 (n=25)	65.1 ± 14.1 (n=52)	65.8 ± 18.0 (n=35)	0.650
Platelets (x 103 uL)	264.0 ± 95.5 (n=143)	303.3 ± 104.2 (n=25)	248.9 ± 92.9 (n=52)	252.3 ± 88.7 (n=35)	0.110
Urea (mmol/L)	10.0 ± 10.3 (n=146)	7.8 ± 3.8 (n=25)	7.2 ± 3.5 (n=52)	7.7 ± 7.1 (n=35)	0.140
Creatinine (umol/L)	123.9 ± 88.1 (n=146)	130.7 ± 115.2 (n=26)	100.0 ± 32.6 (n=52)	107.5 ± 105.0 (n=35)	0.260
Sodium (mmol/L)	135.4 ± 9.8 (n=143)	136.4 ± 4.9 (n=26)	131.9 ± 23.4 (n=52)	138.3 ± 4.0 (n=35)	0.130
Potassium (mmol/L)	4.2 ± 0.5 (n=141)	4.2 ± 0.5 (n=26)	3.9 ± 0.5 (n=52)	4.0 ± 0.5 (n=35)	0.010
Albumin (g/L)	32.2 ± 5. (n=131)	31.9 ± 5.2 (n=23)	35.8 ± 16.4 (n=50)	31.4 ± 4.9 (n=33)	0.060
Normal ALT/AST	107/135 (79.3%)	17/23 (73.9%)	30/50 (60.0%)	23/34 (67.6%)	0.060
Elevated bilirubin	17/129 (13.2%)	4/22 (18.2%)	12/51 (23.5%)	5/31 (16.1%)	0.400
NT-pro-BNP (pg/mL)	5030 ± 8764 (n=122)	7618 ± 16446 (n=23)	6731 ± 13837 (n=50)	4443 ± 6277 (n=30)	0.570
Troponin-T – peak (ng/L)	422 ± 1963 (n=126)	1402 ± 3828 (n=24)	1809 ± 5644 (n=46)	688 ± 1592 (n=33)	0.070
Iron transferrin saturation (%)	20.6 ± 22.0 (n=41)	17.30 ± 12.81 (n=6)	12.40 ± 7.1 (n=15)	13.08 ± 5.81 (n=12)	0.340
Ferritin (mcg/L)	182 ± 188 (n=36)	426 ± 223 (n=6)	233 ± 221 (n=14)	346 ± 366 (n=13)	0.050
Glycated hemoglobin – HBA1c (%)	8.3 ± 2.1 (n=147)	8.7 ± 2.9 (n=24)	6.0 ± 0.3 (n=51)	5.3 ± 0.3 (n=21)	0.001
Lactic acid (mmol/L)	2.3 ± 1.7 (n=56)	2.2 ± 0.8 (n=14)	1.8 ± 0.9 (n=33)	1.9 ± 1.1 (n=15)	0.420
LDL-C (mmol/L)	2.8 ± 1.6 (n=70)	3.8 ± 1.7 (n=19)	2.9 ± 1.3 (n=30)	3.1 ± 1.5 (n=21)	0.050

ACEI/ARB/ARNI, angiotensin-converting enzyme inhibitors/angiotensin II receptor blockers/angiotensin receptor-neprilysin inhibitor; ALT/AST, alanine transaminase/aspartate transaminase; BP, blood pressure; bpm: beats per minute; DM: diabetes mellitus; ECG, electrocardiogram; HFmrEF, heart failure with mildly reduced ejection fraction; HFpEF, heart failure with preserved ejection fraction; HFrEF, heart failure with reduced ejection fraction; HDZ/ISDN, hydralazine/isosorbide dinitrate; JVP, jugular venous pressure; LBBB, left bundle branch block; LDL-C: low-density lipoprotein cholesterol; LVEF, left ventricular ejection fraction; msec: millisecond; NT-pro-BNP, N-terminal pro-brain natriuretic peptide; SpO2: peripheral capillary oxygen saturation; VHD, valvular heart disease.

The left ventricular ejection fraction (LVEF) values ranged between 29.5% and 36.6% across the groups with the highest value reported in patients with known DM. The HF phenotypes according to the LVEF cutoffs did not differ within DNHF categories. Most of the study population had HF with reduced ejection fraction (range across the groups: 70.3 – 92.3%). Almost two-thirds of new and known diabetic patients (69.6% and 76.8%) had ischemic HF etiology, whereas almost half of the pre-diabetics and non-diabetics (47.1% and 48.0%) had non-ischemic etiology. Most of the study population had HF with reduced ejection fraction (70.3 – 92.3%). Patients with new DM tended to have a greater non-ischemic etiology of HF (30.4%), and a higher incidence of HF with reduced ejection fraction (92.3%) than those with known DM (23.2% and 70.3%, respectively). Guideline-directed HF medications during hospital stay did not differ between the study groups, except for the diuretics use which was higher in the new and known diabetic groups (92.3% and 85.7%) than the non- and pre-diabetic groups (71.4% and 75.0%) (**Table 3**). Similarly, laboratory blood tests and guideline-directed HF medications used were comparable within DNHF categories upon hospital discharge. The mean natriuretic peptide levels among the groups ranged from 2780 to 5464 pg/mL upon hospital discharge (**Table 4**).

**Table 3:** Investigations and management during index hospitalization.

Variable	Known DM (n=147)	New DM (n=26)	Pre-DM (n=52)	No DM (n=35)	P-value
ECG and echocardiogram					
LBBB	15/130 (11.5%)	2/20 (10.0%)	5/50 (10.0%)	3/33 (9.1%)	0.970
QRS duration (msec)	104.6 ± 24.1 (n=137)	109.0 ± 21.3 (n=22)	106.6 ± 27.1 (n=51)	101.6 ± 23.0 (n=32)	0.690
LVEF (%)	36.7 ± 17.1 (n=116)	29.5 ± 6.2 (n=22)	30.0 ± 9.3 (n=49)	32.8 ± 11.5 (n=27)	0.020
Heart failure phenotypes					
LVEF < 40% (HFrEF)	97/138 (70.3%)	24/26 (92.3%)	45/51 (88.2%)	25/34 (73.5%)	0.050
LVEF 40 – 49% (HFmrEF)	27/138 (19.6%)	2/26 (7.7%)	4/51 (7.8%)	4/34 (11.8%)	
LVEF ≥ 50% (HFpEF)	14/138 (10.1%)	0/26 (0.0%)	2/51 (3.9%)	5/34 (14.7%)	
Diastolic dysfunction					
Normal	6/84 (7.1%)	1/19 (5.3%)	0/31 (0.0%)	2/19 (10.5%)	0.320
Grade 1	26/84 (31.0%)	10/19 (52.6%)	9/31 (29.0%)	9/19 (47.7%)	
Grade 2	28/84 (28.6%)	4/19 (21.1%)	10/31 (32.3%)	6/19 (31.6%)	
Grade 3	24/84 (28.6%)	4/19 (21.1%)	12/31 (38.7%)	2/19 (10.5%)	
Heart failure etiology					
Ischemic	106/138 (76.8%)	16/23 (69.6%)	26/50 (52.0%)	18/34 (52.9%)	0.003
Non-ischemic	32/138 (23.2%)	7/23 (30.4%)	24/50 (48.0%)	16/34 (47.1%)	
Idiopathic	30/147 (20.4%)	11/26 (42.3%)	11/52 (21.2%)	8/35 (22.9%)	0.106
Etiology details					
Hypertension	116/147 (78.9%)	21/26 (80.8%)	27/52 (51.9%)	19/35 (54.3%)	0.001
Primary VHD	2/147 (1.4%)	2/26 (7.7%)	6/52 (11.5%)	1/35 (2.9%)	0.013
Secondary VHD	4/147 (2.7%)	0/26 (0.0%)	2/52 (3.8%)	1/35 (2.9%)	0.800
Tachycardia-induced	7/147 (4.8%)	1/26 (3.8%)	2/52 (3.8%)	1/35 (2.9%)	0.960
Management during admission					
ACEI/ARB/ARNI	90/147 (61.2%)	17/26 (65.4%)	38/52 (73.1%)	22/35 (62.9%)	0.500
Beta-blockers	122/147 (83.0%)	20/26 (76.9%)	46/52 (88.5%)	29/35 (82.9%)	0.620
Mineralocorticoids	58/147 (39.5%)	12/26 (46.2%)	30/52 (57.7%)	12/35 (34.3%)	0.090
HDZ/ISDN	25/147 (17.0%)	3/26 (11.5%)	3/52 (5.8%)	2/35 (5.7%)	0.100
Diuretics	126/147 (85.7%)	24/26 (92.3%)	39/52 (75.0%)	25/35 (71.4%)	0.050
Loop diuretics	90/147 (61.2%)	13/26 (50.0%)	24/52 (46.2%)	17/35 (48.6%)	0.190
Digoxin	7/147 (4.8%)	2/26 (7.7%)	7/52 (13.5%)	0/35 (0.0%)	0.050
Ivabradine	9/147 (6.1%)	2/26 (7.7%)	4/52 (7.7%)	1/35 (2.9%)	0.810
Antiplatelet therapy	93/147 (63.3%)	19/26 (73.1%)	26/52 (50.0%)	15/35 (42.9%)	0.030
Oral anticoagulation	97/147 (66.0%)	19/26 (73.1%)	42/52 (80.0%)	22/35 (62.9%)	0.190
Warfarin	10/147 (6.8%)	0/26 (0.0%)	10/52 (19.2%)	2/35 (5.7%)	0.010
NOACs	75/147 (51.0%)	16/26 (61.5%)	29/52 (55.8%)	16/35 (45.7%)	0.610
Nitroglycerine	11/147 (7.5%)	4/26 (15.4%)	4/52 (7.7%)	3/35 (8.6%)	0.610
Diabetic medications	132/147 (89.8%)	20/26 (76.9%)	8/52 (15.4%)	2/35 (5.7%)	0.001
Inotrope/vasopressor support	13/147 (8.8%)	0/26 (0.0%)	7/52 (13.5%)	2/35 (5.7%)	0.220
Coronary angiography	72/146 (49.3%)	16/26 (61.5%)	27/51 (52.9%)	16/35 (45.7%)	0.620

ACEI/ARB/ARNI, angiotensin-converting enzyme inhibitors/angiotensin II receptor blockers/angiotensin receptor-neprilysin inhibitor; ALT/AST, alanine transaminase/aspartate transaminase; BP, blood pressure; bpm, beats per minute; DM, diabetes mellitus; ECG, electrocardiogram; HFmrEF, heart failure with mildly reduced ejection fraction; HFpEF, heart failure with preserved ejection fraction; HFrEF, heart failure with reduced ejection fraction; HDZ/ISDN, hydralazine/isosorbide dinitrate; JVP, jugular venous pressure; LBBB, left bundle branch block; LDL-C, low-density lipoprotein cholesterol; LVEF, left ventricular ejection fraction; msec, millisecond; NT-pro-BNP, N-terminal pro-brain natriuretic peptide; SpO2, peripheral capillary oxygen saturation; VHD, valvular heart disease.

**Table 4:** Hospital discharge key laboratory tests, medications, and clinical outcomes.

Variable	Known DM (n=147)	New DM (n=26)	Pre-DM (n=52)	No DM (n=35)	P-value
Blood tests at discharge					
Hemoglobin (g/dL)	11.9 ± 2.1 (n=133)	13.0 ± 2.1 (n=22)	13.2 ± 2.2 (n=47)	12.1 ± 2.3 (n=32)	0.001
Creatinine (umol/L)	121.4 ± 87.7 (n=140)	118.8 ± 101.9 (n=25)	95.6 ± 31.2 (n=51)	102.2 ± 70.1 (n=35)	0.190
Sodium (mmol/L)	135.9 ± 3.8 (n=140)	136.8 ± 3.0 (n=25)	137.3 ± 3.0 (n=51)	137.4 ± 2.4 (n=35)	0.020
Potassium (mmol/L)	4.5 ± 3.4 (n=139)	4.2 ± 0.4 (n=25)	4.2 ± 0.3 (n=50)	4.2 ± 0.3 (n=35)	0.830
NT-pro-BNP (pg/mL)	5030 ± 8764 (n=122)	7618 ± 16446 (n=23)	6731 ± 13837 (n=50)	4443 ± 6277 (n=30)	1.000
Medications at discharge					
ACEI	34/136 (25.0%)	8/24 (33.3%)	22/50 (%44.0)	13/35 (37.1%)	0.080
ARB	46/132 (34.8%)	6/24 (25.0%)	8/50 (16.0%)	7/35 (20.0%)	0.050
ARNI	26/134 (19.4%)	5/24 (20.8%)	12/50 (24.0%)	6/35 (17.1%)	0.870
Beta-blockers	130/140 (92.9%)	24/25 (96.0%)	47/50 (94.0%)	30/35 (85.7%)	0.410
Mineralocorticoids	76/137 (55.5%)	13/24 (54.2%)	35/49 (71.4%)	17/35 (48.6%)	0.150
In-hospital outcomes					
In-hospital all-cause death	4/147 (2.7%)	0/26 (0.0%)	2/52 (3.8%)	0/35 (0.0%)	0.550
Cardiovascular death	4/147 (2.7%)	0/26 (0.0%)	2/52 (3.8%)	0/35 (0.0%)	0.550
Time to death from index admission (days)	62.3 ± 73.2 (n=4)	–	37.0 ± 5.7 (n=2)	–	0.670
Length of hospital stay (days)	17.5 ± 67.6 (n=142)	18.9 ± 18.2 (n=26)	18.2 ± 22.3 (n=52)	10.9 ± 10.2 (n=33)	0.910
Readmission outcomes					
Cardiovascular readmissions	11/147 (7.5%)	1/26 (3.8%)	7/52 (13.5%)	1/35 (2.9%)	0.250
30-day readmission	0 (0%)	2 (7.7%)	2 (3.8%)	0 (0%)	–
Time to first hospitalization (months)	6.4 ± 7.8	10.0 ± 0.0	1.7 ± 1.1	4.9 ± 0.0	–
Total number of readmissions (days)	16.0 (1.0 – 3.0)	1.0	12.0 (1.0 – 4.0)	1.0	–

ACEI, angiotensin-converting enzyme inhibitors; ARB, angiotensin II receptor blockers; ARNI, Angiotensin receptor-neprilysin inhibitor; DM, diabetes mellitus; NT-pro-BNP, N-terminal pro-brain natriuretic peptide.

### Clinical outcomes

A total of six patients died during the hospital stay due to cardiovascular reasons, four of them were with known DM and the remaining two were prediabetics. The length of hospital stay of the study population ranged from 10.9 to 18.9 days and readmission rates ranged from 2.9% to 13.5% without a significant difference between the study groups. Only a total of four patients were readmitted within 30 days of hospital discharge and the time to first hospitalization was 1.7 – 6.4 months (**Table 4**).

### Incidence of diabetes post-discharge

Among the 31 patients with prediabetes who had a subsequent repeated HbA1c test post discharge, 23 of them (42.0%) developed DM with a mean HbA1c of 8.0% over a mean of 26 months. In the non-diabetic group, 23 patients had documented HbA1c values repeated after discharge, in which only two (8.7%) had HbA1c values of 6.5% or more. One patient had an HbA1c value of 6.7% at 64 months and the other one had an HbA1c value of 11.0% at 29 months follow-up.

## Discussion

This retrospective cohort study from the nationwide heart failure registry revealed that 10% of patients admitted with DNHF had newly diagnosed DM and nearly one-third of the total patient population exhibited previously unrecognized dysglycemia. This highlights an important burden of undiagnosed metabolic disorders in patients presenting with acute HF. The mean HbA1c for patients with newly detected DM was 8.33%. The presence or absence of DM or prediabetes was not associated with any differences in clinical outcomes between the groups of DNHF patients including in-hospital all-cause mortality, cardiovascular death, length of hospital stay, 30-day readmission to the hospital or any hospital readmission due to cardiovascular causes within the study period. A further 42% of those with prediabetes who had follow-up screening performed for DM after discharge were subsequently diagnosed with DM over a mean of 26 months.

Patients with newly diagnosed DM were predominantly male and exhibited fewer traditional cardiovascular comorbidities, such as hypertension, coronary artery disease, chronic kidney disease, compared to those with known DM. Despite this, upon presentation they had the highest prevalence of HF with reduced ejection fraction amongst the groups. This supports the prior hypothesis that the pathophysiology of unrecognized DM leading to HF is complex, and that DM may contribute to the development of HF through more direct mechanisms independent of traditional risk factors. Those mechanisms include insulin resistance, hyperglycemia-induced oxidative stress, advanced glycation end-products deposition in the myocardium, lipotoxicity, and microvascular rarefaction; processes that collectively can impair myocardial contractile function, promote fibrosis, leading to both structural and functional cardiac remodeling (5).

Conversely, there is growing evidence that HF may not only be a consequence of DM but also a precursor to it. This may occur by prompting chronic systemic inflammation, neuro-hormonal activation, and sympathetic overdrive response to the impaired peripheral blood flow; all of which can lead to insulin resistance (6, 10). A large nationwide cohort study in Denmark found that after a hospitalization for DNHF, 10% of the population who were nondiabetic at baseline were found to have developed a new-onset DM during a mean follow-up of 3.9 years (10). This risk appeared to be clinically relevant in our study population as well. In the prediabetic group, 42% progressed to overt DM over an average of 26 months after index admission for DNHF as compared to 8.7% in the non-diabetic group who developed subsequent DM. Further prospective studies are needed to assess the relative risk of DNHF on development of DM. Although the progression rate to overt DM in our prediabetic group was notable, it must be also recognized that in our cohort, many demonstrated elevated cardiovascular risk profiles at baseline namely hypertension and dyslipidemia, all of which may also predispose to development of DM later (11). These results merely reflect the importance of longitudinal metabolic surveillance post-discharge from an acute HF admission regardless of their glycemic status at presentation.

Epidemiological data in the literature suggests that there may be a latency period between DM onset and development of HF. In the Atherosclerosis Risk in Communities (ARIC) cohort, over a median follow-up of 22.5 years, the risk of incident HF increased by 17% with each 5-year increment in diabetes duration with highest risk (nearly threefold) was in those with DM ≥ 15 years (12). Similarly, pooled analysis of the DAPA-HF and DELIVER trials showed that nearly two thirds of their HF cohorts had DM >5 years with 38.3% of patients having DM > 11 years (13). Moreover, individuals with early-onset DM (<40 years) have been shown to carry a twofold age-adjusted relative risk for incident HF compared to a later-onset of DM (14). This data, mainly from studies on chronic HF, points towards the plausibility that in our cohort, many patients may have been undiagnosed for several years, allowing progressive metabolic and myocardial changes to occur. This reflects a missed opportunity to decrease their risk of developing DNHF and reiterates the need for early metabolic surveillance in patients at risk for HF. Further prospective studies are needed to explore the median duration between detection of diabetes and admission for DNHF.

In our study, we found no statistically significant differences in in-hospital mortality, readmission rates, or length of stay across the glycemic spectrum in patients admitted with DNHF. The short-term clinical outcomes in the available literature are mixed. Several large United States-based HF registries (such as ADHERE, OPTIMIZE-HF and Get With The Guidelines-HF), have demonstrated no significant association between Type 2 DM and in-hospital mortality (15–18) whereas the European Society of Cardiology HF Long-Term Registry showed that Type 2 DM was independently associated with a higher risk of in-hospital mortality in acute HF admissions (19). The differences in these studies may be attributed to variations in patient population, and severity of DM. Similarly, the lack of significant differences in the clinical outcomes in our cohort may be partially explained by the relatively modest glycemic elevations at baseline. Although the mean observed HbA1c of 8.5% in the known DM group and 8.3% in the newly diagnosed group are significantly above the diagnostic thresholds, they were not exceptionally high, which may not have translated into immediate clinical deterioration or increased re-hospitalization in the immediate post discharge period. Despite the inconsistencies in results of short-term outcomes in the literature, long-term data confirms that patients with diabetes experience worse prognoses than their nondiabetic counterparts which highlights the importance of ongoing surveillance and proactive glycemic control in the management of HF when DM is recognized.

### Study limitations

The strength of our study is that it is from a registry that was conducted at the single tertiary HF center which serves the entire country providing a comprehensive overview of the interplay between DM and DNHF across the entire population. All peripheral hospitals also share the same electronic health record system which decreased the possibility of missing patients with DNHF admissions. However, certain key limitations, that should be acknowledged, include the study’s retrospective design which led to a small sample size in certain groups and may have potentially limited the statistical power for outcome comparisons. The reliance on HbA1c solely for the diagnosis of DM must be highlighted, since stress-induced hyperglycemia without elevated HbA1c levels was not classified as DM. Additionally, the lack of detailed data on antidiabetic medications use limits the ability to assess the impact of specific therapies on HF outcomes. The decision to repeat HbA1c on patient’s follow-up was left to the treating physician and was not protocolized which led to some patients having missing readings of follow-up HbA1c which limits conclusions regarding the control of DM and the development of new DM during the follow-up period. The results of our study may not be generalizable to other populations given the unique demographic composition of Qatar which tends to underrepresent Western and European populations. Finally, impact of DM on HF outcomes that occurred during the study period were captured and not beyond that. Future studies should be conducted in a prospective fashion to further elucidate the relationship between newly diagnosed DM and HF outcomes, as well as to evaluate the effectiveness of early glycemic control interventions in this population. Increasing the sample size in each group in future studies will also improve the statistical power for outcome comparisons. Results of further studies will add to the growing concept of primary prevention of HF by early detection and management prior to admission for DNHF.

## Conclusion

Our study highlights a notable prevalence of undiagnosed dysglycemia (DM and prediabetes) among patients admitted with DNHF however without a difference in in-hospital and early discharge outcomes between the diabetic and non-diabetic groups. DNHF served as the initial manifestation of diabetes or prediabetes in one third of our cohort. These findings reveal the critical importance of routine metabolic screening during index admission for DNHF, as early identification of glucose intolerance offers an opportunity for a timely intervention. Furthermore, the high prevalence of undiagnosed metabolic abnormalities in the DNHF population warrants the need for a proactive diabetes screening as a strategy for the primary prevention of HF.

## Data Availability

The raw data supporting the conclusions of this article will be made available by the authors upon request, without undue reservation after seeking the relevant institutional approvals.
